# The Response of Ruminal Microbiota and Metabolites to Different Dietary Protein Levels in Tibetan Sheep on the Qinghai-Tibetan Plateau

**DOI:** 10.3389/fvets.2022.922817

**Published:** 2022-06-29

**Authors:** Xungang Wang, Tianwei Xu, Xiaoling Zhang, Na Zhao, Linyong Hu, Hongjin Liu, Qian Zhang, Yuanyue Geng, Shengping Kang, Shixiao Xu

**Affiliations:** ^1^Northwest Institute of Plateau Biology, Chinese Academy of Sciences, Xining, China; ^2^University of Chinese Academy of Sciences, Beijing, China; ^3^State Key Laboratory of Plateau Ecology and Agriculture, Qinghai University, Xining, China

**Keywords:** Tibetan sheep, rumen, microbiota, metabolomics, dietary protein

## Abstract

Ruminal microbiota and metabolites play crucial roles in animal health and productivity. Exploring the dynamic changes and interactions between microbial community composition and metabolites is important for understanding ruminal nutrition and metabolism. Tibetan sheep (*Ovis aries*) are an important livestock resource on the Qinghai-Tibetan Plateau (QTP), and the effects of various dietary protein levels on ruminal microbiota and metabolites are still unknown. The aim of this study was to investigate the response of ruminal microbiota and metabolites to different levels of dietary protein in Tibetan sheep. Three diets with different protein levels (low protein 10.1%, medium protein 12.1%, and high protein 14.1%) were fed to Tibetan sheep. 16S rRNA gene sequencing and gas chromatography coupled with time-of-flight mass spectrometry (GC-TOF-MS) were used to study the profile changes in each group of ruminal microbes and metabolites, as well as the potential interaction between them. The rumen microbiota in all groups was dominated by the phyla Bacteroidetes and Firmicutes regardless of the dietary protein level. At the genus level, *Prevotella_1, Rikenellaceae_RC9_gut_group* and *Prevotellaceae_UCG-001* were dominant. Under the same forage-to-concentrate ratio condition, the difference in the dietary protein levels had no significant impact on the bacterial alpha diversity index and relative abundance of the major phyla and genera in Tibetan sheep. Rumen metabolomics analysis revealed that dietary protein levels altered the concentrations of ruminal amino acids, carbohydrates and organic acids, and significantly affected tryptophan metabolism (*p* < 0.05). Correlation analysis of the microbiota and metabolites revealed positive and negative regulatory mechanisms. Overall, this study provides detailed information on rumen microorganisms and ruminal metabolites under different levels of dietary protein, which could be helpful in subsequent research for regulating animal nutrition and metabolism through nutritional interventions.

## Introduction

Tibetan sheep are the most economically important domestic animals on the Qinghai-Tibetan Plateau (QTP), providing native Tibetan herders with meat, wool and milk (1). On the QTP, Tibetan sheep adapt well to plateau environments and poor feeding conditions, and they mainly search for forage in the alpine meadow (2, 3). Nonetheless, the QTP environment is extremely harsh, with heavy snowfalls during the long cool-season from November to May, with average temperatures ranging from −5 to −15°C. Climate conditions and fluctuations directly affect forage yield and quality (particularly crude protein content), and herbage biomass and nutritional status are insufficient to meet the daily nutritional requirements of grazing animals (4–6). A previous study showed that Tibetan sheep suffered serious live-weight loss (−20.54%) under traditional pastoral herding, resulting in severe economic loss during the cold season (7). In addition to growth performance and economic benefits, cold season grazing also reduces growth hormone levels and damages the immune defense system of Tibetan sheep (8). Therefore, scientific management and rearing are particularly important in Tibetan sheep production.

The rumen is the primary organ system for nutrient digestion and absorption in ruminants, and it contains abundant microbiota and metabolites. Rumen microorganisms play an important role in the fermentation of plant fibers and polysaccharides (9, 10). Previous studies have shown that rumen microbial community structure and function are influenced by different factors, such as host breed, sex, diet and external environment (11–13). As one of the most important factors, dietary nutrition can change the relative abundance of dominant bacterial groups (e.g., Bacteroidetes, Firmicutes, Proteobacteria) and metabolic functions (e.g., carbohydrate, amino acid, and energy metabolism) (14–16). Metabolomics is a newly emerging field, following the application of genomics and transcriptomics based on detection techniques and includes nuclear magnetic resonance (NMR), gas chromatography-mass spectrometry (GC-MS), and liquid chromatography-mass spectrometry (LC-MS) (17). Metabolite profiles include a huge array of organic endogenous metabolites that play a vital role in nutrient regulation in animals. Previous studies have reported that ruminal lipids, amino acids and carbohydrates change significantly by changing the dietary forage-to-concentrate ratio (18, 19).

Dietary protein level is considered an essential factor that affects the growth, development and health conditions of animals (20, 21). Proteins fed to ruminants are degraded by microbes into peptide-bound amino acids and free amino acids for microbial protein synthesis (22). However, the effect of dietary protein levels on the ruminal microbiota and metabolites of Tibetan sheep and the interaction between ruminal microbiota and metabolites remain unclear. Recent rapid developments and applications in multi-omics technology have been devoted to gaining a better understanding of the ruminal ecosystem, especially the relationships among the microbiota, metabolites and host (23, 24).

In the current study, we hypothesize that dietary protein levels would influence rumen microorganisms and ruminal metabolites in Tibetan sheep. 16S rRNA sequencing and GC-TOF-MS were used to determine the effects of three different dietary protein levels on the profiles of the ruminal microbiota and metabolites in Tibetan sheep. Furthermore, the potential relationships between the ruminal microbiota and metabolites were explored.

## Materials and Methods

### Ethics Statement

The animal experiments in this study were approved by the Experimental Animal Use Ethics Committee of the Northwest Institute of Plateau Biology, Chinese Academy of Sciences (Approval No. NWIPB20160302).

### Experimental Design and Sample Collection

A detailed description of the experimental design has been previously provided (25). The experiment was conducted at the Haibei Demonstration Zone of Plateau Modern Ecological Husbandry Science and Technology in Qinghai Province (China) (36°55′N, 100°57′E, altitude at 3,100 m). A total of eighteen 1-yr-old healthy castrated Tibetan sheep with similar initial body weight (BW: 31.71 ± 0.72 kg) were randomly assigned to three different dietary treatment groups, with each group containing 6 sheep. All those sheep were bred in the same demonstration zone and under the same feeding management practices before the experiment. The protein levels of the three different diets were low protein 10.1% (LP), medium protein 12.1% (MP) and high protein 14.1% (HP). Diets were designed according to the National Research Council guidelines (26) (Table 1). The sheep were fed with the mixed diet twice daily at 08:00 and 17:00. The experiment was lasted 105 days after 15 days of adaptation to the experimental diets and all sheep were provided with water ad libitum. At the end of the experiment, rumen fluid samples were collected before the morning feeding using an oral stomach tube and placed in frozen tubes to avoid contamination. The samples were immediately frozen in liquid nitrogen, and stored at −80°C for microbiome and metabolome analysis.

**Table 1:** Ingredients and nutrient levels of the experimental diets with three different protein levels (on a dry matter basis).

**Item**	**Diet**		
	**LP**	**MP**	**HP**
**Ingredients, g/kg**			
Oat hay	500	500	500
Corn grain	210	165	120
Wheat grain	135	120	105
Wheat bran	70	75	80
Soybean meal	35	55	75
Rapeseed meal	25	60	95
NaCl	5	5	5
CaHPO_4_·2H_2_O	3	3	3
Bentonite	5	5	5
CaCo_3_	4.5	4.5	4.5
NaHCO3	2.5	2.5	2.5
Premix ^a^	5	5	5
**Nutrient levels** ^**b**^			
CP (%)	10.1	12.1	14.1
ME (MJ/kg)	10.1	10.1	10.1
EE (%)	2.7	2.8	2.9
NDF (%)	37.5	38.5	39.5
ADF (%)	19.1	20.1	21.1
Ca (%)	0.6	0.7	0.7
P (%)	0.4	0.5	0.5

a *Premix provided per kg of feed: Vitamin A, 50,000 IU; Vitamin D3, 12,500 IU; Vitamin E, 1,000 IU; Cu, 250 mg; Fe, 12,000 mg; Zn, 1,000 mg; Mn, 1,000 mg and Se, 7.5 mg*.

b *ME, metabolizable energy = total digestible nutrients ×0.04409 ×0.82, according to the National Research Council; CP, crude protein; EE, ether extract; NDF, neutral detergent fiber; ADF, acid detergent fiber*.

### Microbiome Composition Analysis

Total genomic DNA was extracted from the rumen fluid samples using the bacterial genomic DNA extraction kit from TIANamp (TIANGEN, Beijing, China). The 16S rRNA gene targeting the V3-V4 region was amplified from the total genomic DNA and sequenced using the Illumina NovaSeq 6000 platform. After sequencing, the raw sequences were analyzed using USEARCH 10.0 and scripts written by Liu et al. (27). The quality of the paired-end Illumina reads was checked using FastQC v.0.11.5 (28) and processed using USEARCH. Unique reads were denoised into ASVs using unoise3 in USEARCH (29). A feature table was generated using VSEARCH (30). The SILVA v123 (31) database was used to classify the taxonomy of the representative sequences, and the plastids and non-bacteria were removed. Alpha diversity indices, including richness and the Shannon index, were calculated. For beta diversity, variations in microbial composition among the three different groups were investigated using constrained PCoA (CPCoA).

### Metabolomics Data Analysis

Rumen fluid samples were centrifuged at 4 °C for 5 min at 10,000 rpm and transferred into a 1.5 ml tube, and pre-cold methanol with 10 μl internal standard 2-Chloro-L-phenylalanine was added. After centrifugation, 200 μl of supernatant was transferred to a fresh tube. Fifty microliters of each sample were removed and combined to prepare a quality control sample. After evaporation in a vacuum concentrator, 30 μl of methoxyamination hydrochloride was added and derivatized with 40 μl of BSTFA reagent at 70 °C for 1.5 h. All samples were then analyzed by GC-TOF-MS. The GC-TOF-MS analysis was performed using an Agilent 7890 GC-TOF-MS. The system used a DB-5MS capillary column (30 m ×250 μm ×0.25 μm). Chroma TOF (V 4.3x, LECO) and the LECO-Fiehn Rtx5 database were used for the raw data analysis, including peak extraction, baseline adjustment, deconvolution, alignment and integration. Finally, peaks detected in less than half of the quality control samples or RSD > 30% in the quality control samples were removed.

The resulting data were imported into software SIMCA 14.1 software (Umetrics, Umea, Sweden) for orthogonal projections to latent structures-discriminant analysis (OPLS-DA). Differential metabolites were identified by combining the VIP values obtained from the OPLS-DA analysis and t-test (VIP > 1.5 and *p* < 0.05). Differential metabolites were identified and validated using the Human Metabolome Database (HMDB; https://hmdb.ca/) and Kyoto Encyclopedia of Genes and Genomes (KEGG; https://www.kegg.jp/). The data analysis tool MetaboAnalyst 5.0 (https://www.metaboanalyst.ca/) was used to view the metabolic pathway distribution and enrichment of the differential metabolites.

### Correlations Between Microbial Communities and Rumen Metabolites

Rumen metabolites with VIP > 1.5, *p* < 0.05, and the top 10 microbial genera were used for interactive analysis. Spearman's rank correlations and *p*-value were calculated using the GenesCloud tool, a website for microbial analysis (http://www.genescloud.cn).

## Results

### Sequencing and Diversity Estimates of Rumen Microbiomes

**Figure 1:**
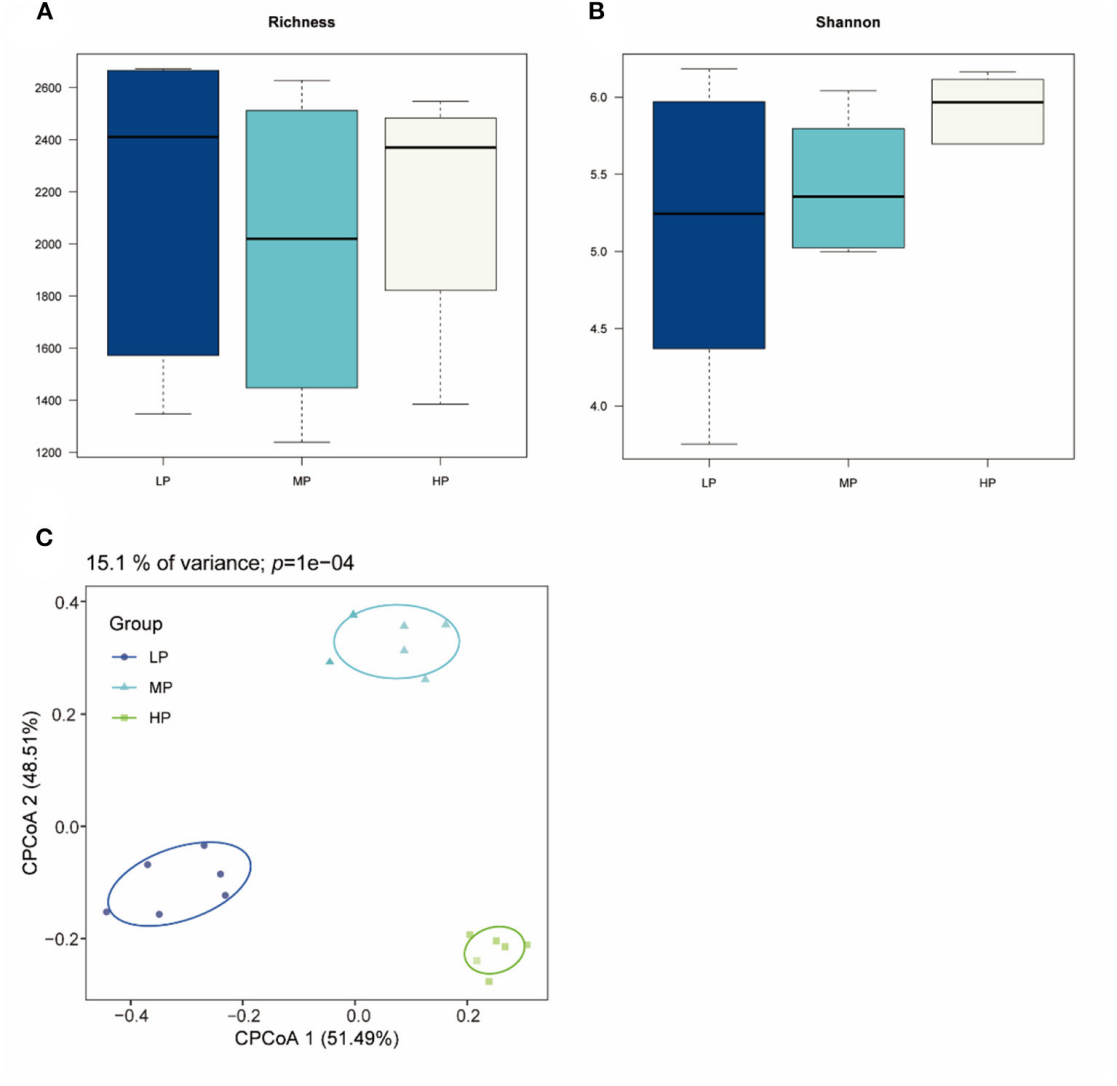
Bacterial diversity of rumen fluid samples among three different groups. **(A)** Richness index; **(B)** Shannon index; **(C)** CPCoA plot based on ASVs.

Totally 2,144,910 raw reads were obtained for the bacterial 16S rRNA genes from 18 rumen fluid samples using the sequencing procedure. After quality control, 2,046,323 high-quality reads were obtained (average of 85,263 reads per sample). A total of 3,921 ASVs were produced based on the results. The statistics of the bacterial alpha diversity indices (richness and Shannon index) for each sample were calculated, and the results are shown in Figures 1A,B. Richness and Shannon values were not significant among the three groups (*p* > 0.05). The result of beta diversity based on the CPCoA showed that the rumen microbiota of Tibetan sheep clustered three distinct parts, and these three groups were largely separated from each other with 15.1% of the variance (*p* = 0.0001) (Figure 1C).

### Bacterial Community Compositions

**Figure 2:**
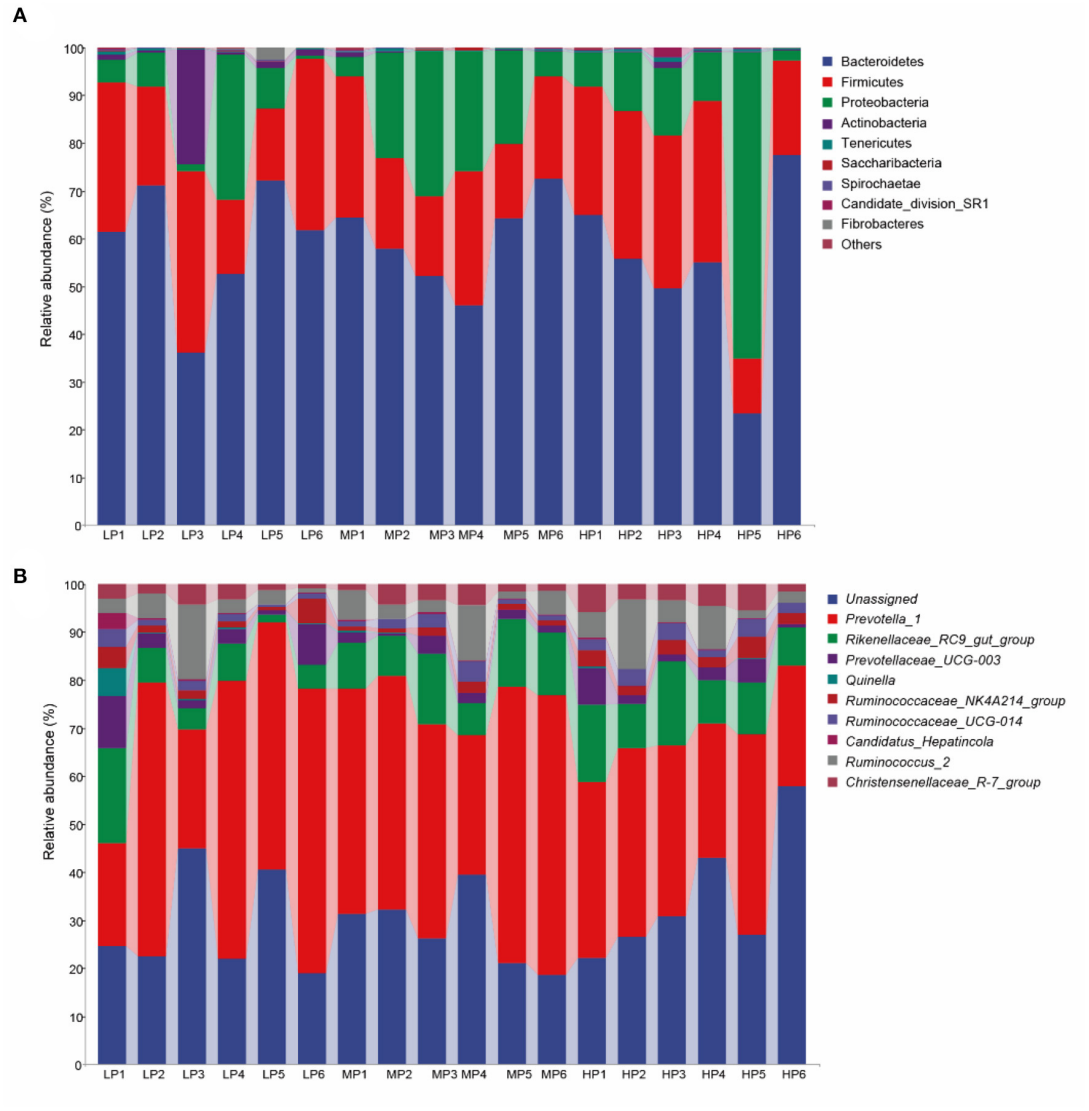
Microbial composition of rumen fluid samples at the **(A)** phylum and **(B)** genus level.

At the taxonomic level, 16 bacterial phyla, 26 classes, 37 orders, 56 families, and 163 genera were detected in the rumen microbiota of Tibetan sheep. At the phylum level (Figure 2A), Bacteroidetes (54.37–59.58%), Firmicutes (21.68–26.10%), Proteobacteria (8.81–18.46%), Actinobacteria (0.27–4.78%), Tenericutes (0.23–0.36%), Spirochaete (0.07–0.15%), Saccharibacteria (0.06–0.10%) and Candidate_division_SR1 (0.02–0.35%) were the dominant bacteria. Among these phyla, Bacteroidetes and Firmicutes had the highest relative abundances in all three groups. However, no significant between-group differences in relative abundance were detected at the phylum level. At the genus level (Figure 2B), *Prevotella_1* (21.10–32.38%), *Rikenellaceae_RC9_gut_group* (4.94–7.52%), *Prevotellaceae_UCG-001* (2.18–2.47%), *Prevotellaceae_UCG-003* (1.28–2.61%) and *Christensenellaceae_R-7_group* (1.45–2.45%) were the dominant bacteria. In addition, the relative abundance of these dominant bacterial genera was also not significantly affected by dietary protein levels (*p* > 0.05).

### Identification and Quantification of GC-TOF-MS Metabolites in the Rumen

In total, 411 valid peaks were identified in 18 rumen fluid samples. After rigorous quality control and identification, 189 metabolites, including organic acids and derivatives, organoheterocyclic compounds, organic oxygen compounds, benzenoids, organic oxygen compounds, lipids, lipid-like molecules and benzenoids, were obtained from the metabolomics library of the three groups, which shared the same metabolite categories.

**Figure 3:**
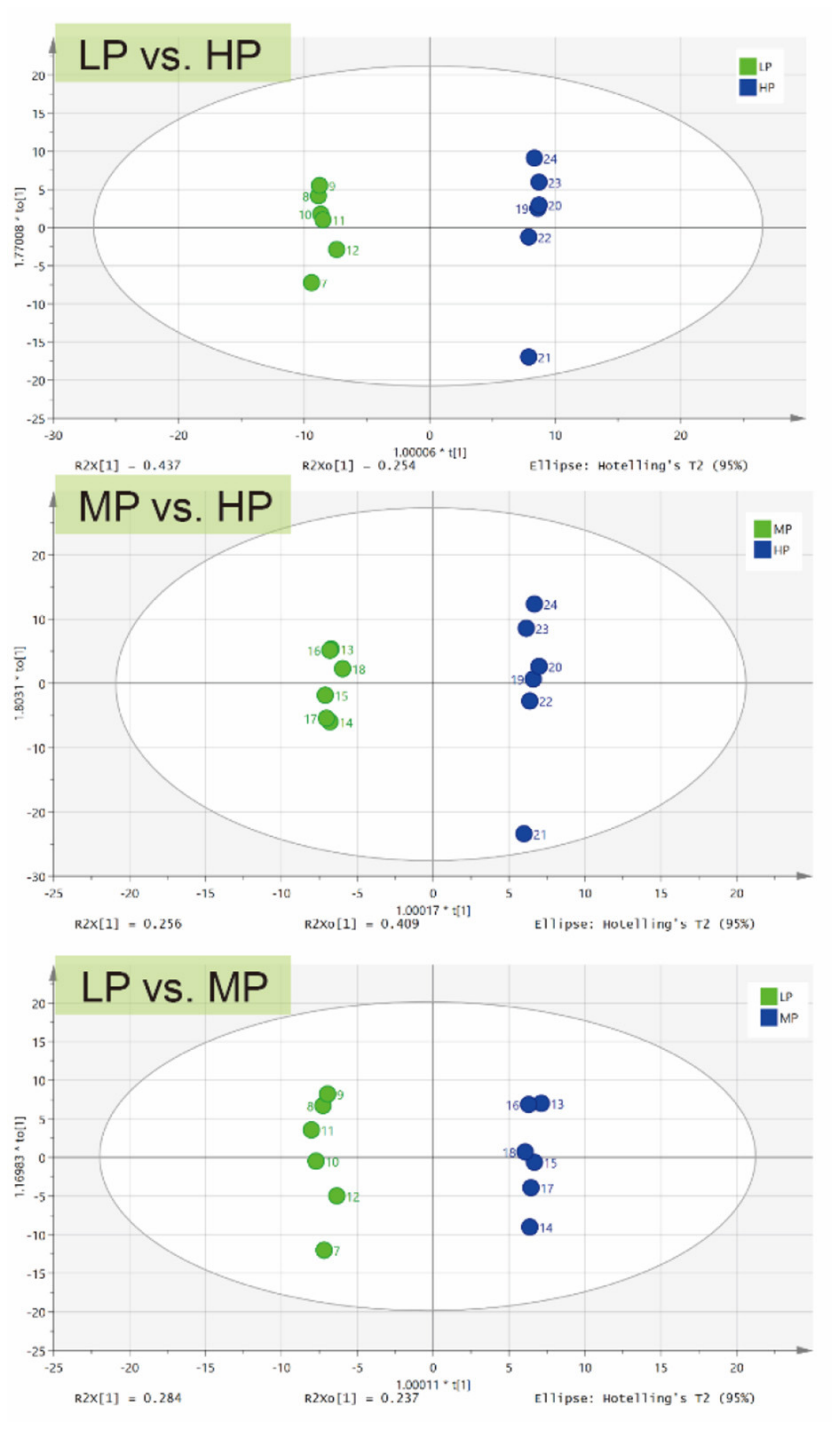
OPLS-DA of ruminal metabolites between different groups.

For further analysis, OPLS-DA was conducted to characterize the differences in rumen metabolic profiles between the different groups. The parameters for the assessment of the OPLS-DA model in differentiating the three groups is represented by validation plots (Figure 3). The corresponding R^2^Y values of the OPLS-DA model for LP vs. HP, MP vs. HP and LP vs. MP were 0.954, 0.98 and 0.835, respectively. This indicates that this model can be used to identify differences between the groups. OPLS-DA results also showed that these groups had distinctly different metabolite compositions.

### Rumen Metabolomic Profiles

Based on the statistical analysis results and the VIP values obtained from OPLS-DA, 17 metabolites (*p* < 0.05, and VIP > 1.5) were found to be significantly different in the comparisons of HP vs. LP, MP vs. LP, and HP vs. MP. Among these, three metabolites were classified into benzenoids (super class level; the same as below); four were classified into lipids and lipid-like molecules; three were classified into organic acids and derivatives; three were classified into organic oxygen compounds; three were classified into oganioheterocyclic compounds; and one was classified into homogeneous non-metal compounds.

**Figure 4:**
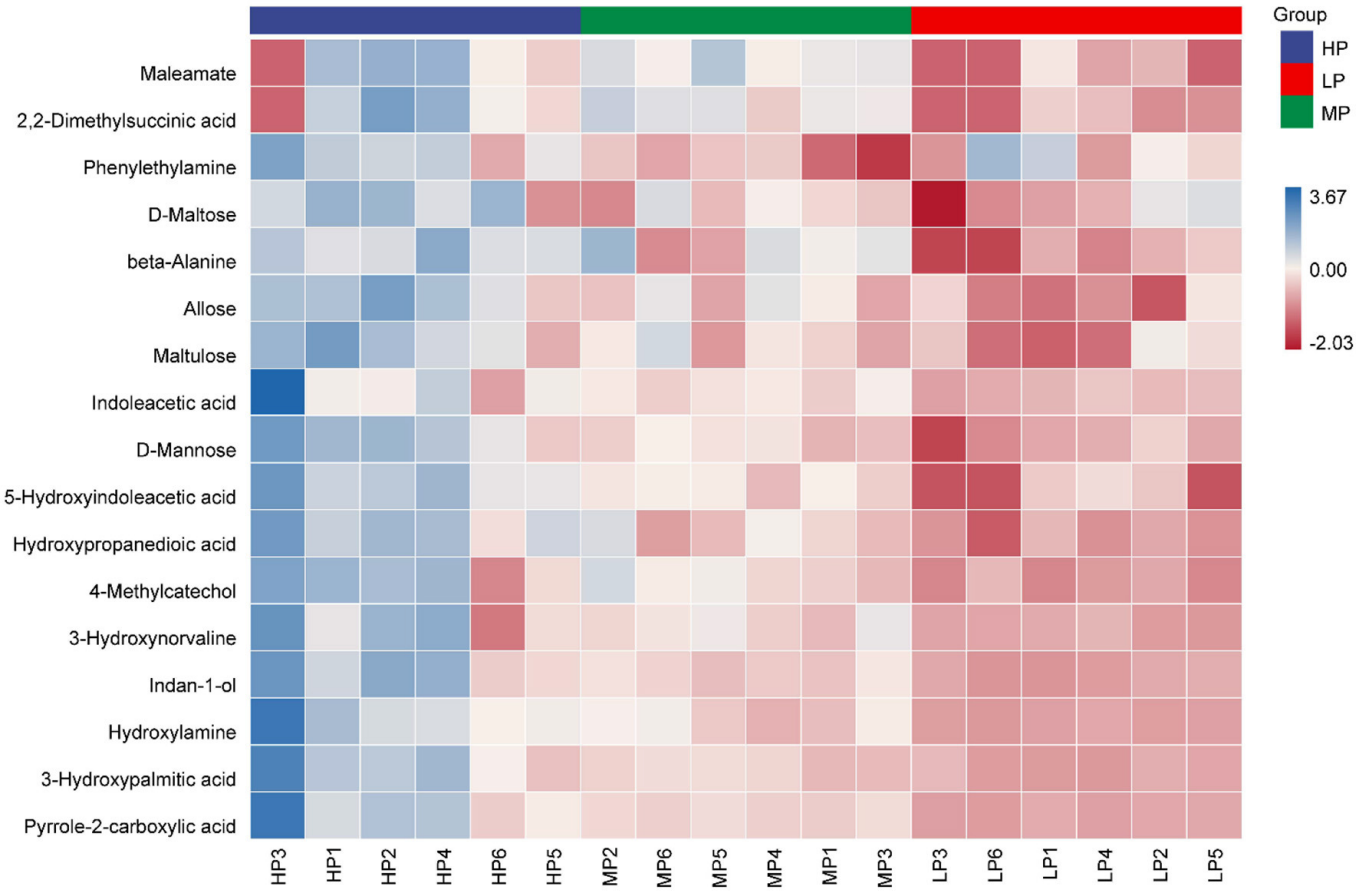
Heatmap of differential metabolites in rumen fluid samples between different groups.

With an increase in dietary protein level, 17 metabolites showed an increase (Figure 4; Supplementary Table 1). Compared with the LP group, three metabolites (beta-Alanine, Hydroxypropanedioic acid and 5-Hydroxyindoleacetic acid) in the HP group increased significantly (VIP > 1.5, *p* < 0.05). Compared with the MP group, six metabolites (D-Mannose, Allose, Phenylethylamine, Indan-1-ol, D-Maltose and Maltulose) in the HP group increased significantly (VIP > 1.5, *p* < 0.05). Compared with the LP group, eight metabolites (Pyrrole-2-carboxylic acid, Indoleacetic acid, 3-Hydroxypalmitic acid, 2,2-Dimethylsuccinic acid, Maleamate, 3-Hydroxynorvaline, Hydroxylamine and 4-Methylcatechol) in the MP group increased significantly (VIP > 1.5, *p* < 0.05).

### Metabolic Pathways of Differential Metabolites

**Figure 5:**
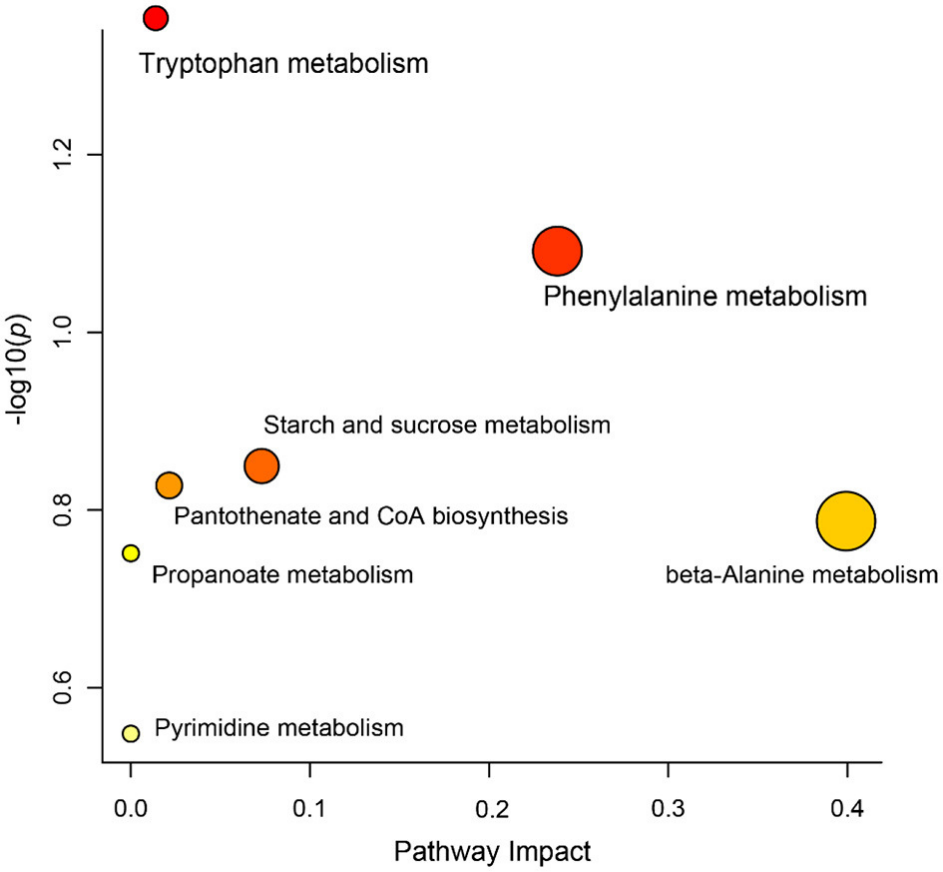
KEGG pathways based on the differential metabolites identified in different groups.

Differential metabolites in rumen fluid samples from the three groups were analyzed using MetaboAnalyst 5.0 software to reveal their association with metabolic pathways (Figure 5). According to KEGG pathway identification, seven pathways (tryptophan metabolism, phenylalanine metabolism, starch and sucrose metabolism, pantothenate and CoA biosynthesis, beta-alanine metabolism, propanoate metabolism and pyrimidine metabolism) were identified. Tryptophan metabolism was the most altered metabolic pathway among the three groups (*p* < 0.05).

### Relationship Between the Ruminal Microbiome and Metabolome

**Figure 6:**
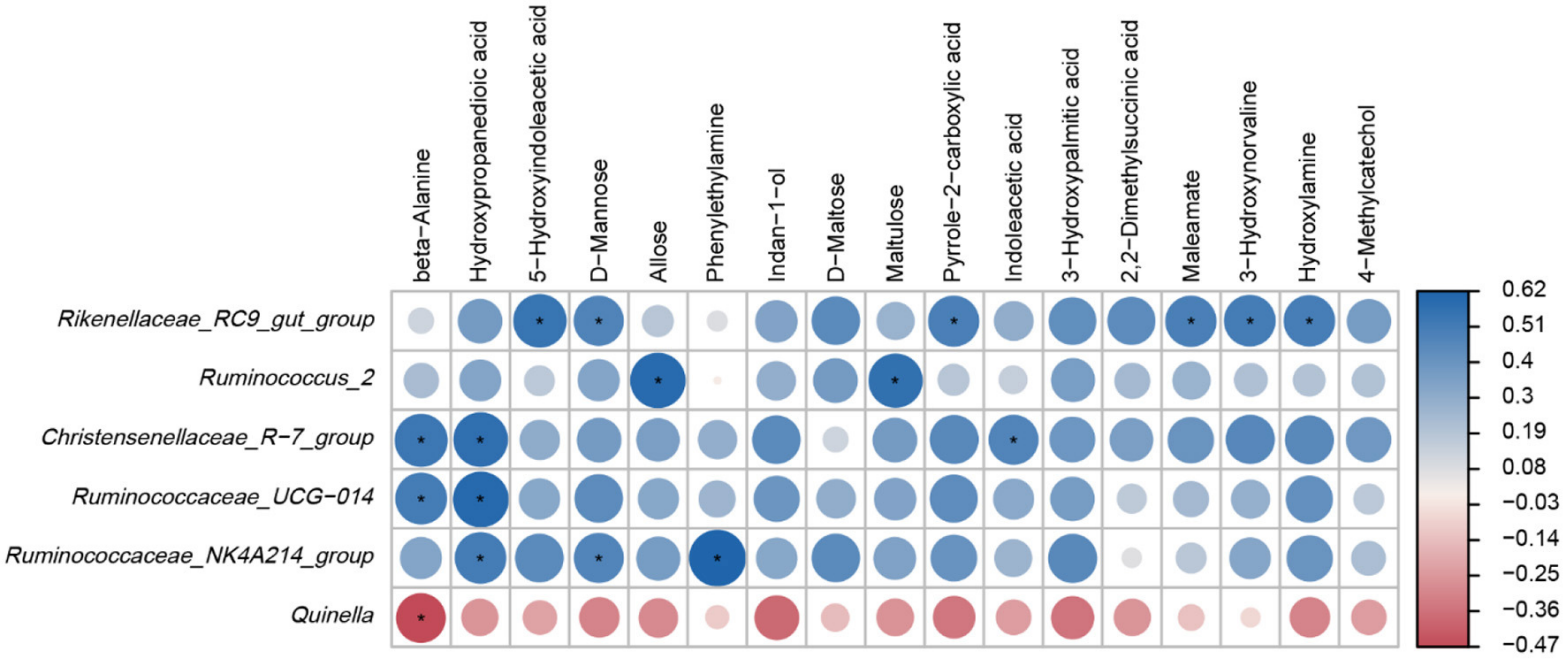
Heatmap of Spearman correlations between main genera and differential metabolites. *p < 0.05.

Based on the results of Spearman correlation coefficients and *p* values, and clear positive and negative correlations were detected between the main ruminal microbiota and differential metabolites (Figure 6). For example, *Rikenellaceae_RC9_gut_group* was positively associated with 5-Hydroxyindoleacetic acid, D-Mannose, Pyrrole-2-carboxylic acid, Maleamate, 3-Hydroxynorvaline and Hydroxylamine (*p* < 0.05). *Ruminococcus_2* was positively correlated with Allose and Maltulose levels (*p* < 0.05). *Christensenellaceae_R-7_group* was positively correlated with beta-Alanine, Hydroxypropanedioic acid and Indoleacetic acid (*p* < 0.05). *Ruminococcaceae_UCG-014* was positively correlated with beta-Alanine and Hydroxypropanedioic acid levels (*p* < 0.05). *Ruminococcaceae_NK4A214_group* was positively correlated with Hydroxypropanedioic acid, D-Mannose and Phenylethylamine (*p* < 0.05) while *Quinella* was negatively associated with beta-Alanine levels (*p* < 0.05).

## Discussion

Rumen bacterial communities and metabolites play important roles in the growth, development and organismal health of ruminants (32, 33). The objective of this study was to investigate the effects of different dietary protein levels with the same metabolizable energy level on ruminal microbiota and metabolites in Tibetan sheep using 16S rRNA sequencing and GC-TOF-MS and to detect the potential relationships between ruminal bacteria and metabolites.

In this study, the dietary protein levels did not have a significant influence on alpha diversity (based on the richness and Shannon index) or the relative abundance of the main bacterial phyla and genera. A previous study showed that under the same forage-to-concentrate ratio, the alteration of dietary energy level had no significant influence on the alpha diversity and bacterial community structure in Holstein heifers (34). This finding is consistent with research that shows that changing one nutrient content of the diet (e.g., protein or energy) under the same food ingredients was not sufficient to cause a strong fluctuation in the ruminal microbiota. Several studies (18, 35–37) showed that the dietary forage-to-concentrate ratio under the same food ingredients was the most critical environmental factors shaping rumen microbial structures and composition. In our study, Bacteroidetes, Firmicutes, and Proteobacteria were the predominant bacterial phyla in the rumen of Tibetan sheep, and their relative abundances did not show significant impact among the three groups. Bacteroidetes and Firmicutes play a critical role in microbial ecology and are involved in the decomposition of fibrous and non-fibrous diets (38, 39). The phylum Proteobacteria is the largest phylum of bacteria, including many pathogenic bacteria, such as *Escherichia coli, Salmonella, Vibrio cholerae* and *Helicobacter pylori*. Although the relative abundance of Proteobacteria is much lower compared to the Bacteroidetes and Firmicutes, it still plays an important role in rumen metabolism, such as biofilm formation and digestion of soluble carbohydrates (40). Besides, Bacteroidetes and Proteobacteria are the two major N-metabolizing microbial communities (41). Liu et al. (42) demonstrated that Tibetan sheep fed high-concentrate (45–60%) diets significantly increased the relative abundance of Bacteroidetes and reduced the Proteobacteria to adapt to a diet containing more non-fibrous carbohydrates and polysaccharides. At the genus level, the dominant genera (e.g., *Prevotella_1, Rikenellaceae_RC9_gut_group, Prevotellaceae_UCG-001, Prevotellaceae_UCG-003* and *Christensenellaceae_R-7_group*) were also not influenced by dietary protein levels. *Prevotella* has been found predominant in the rumen of sheep and can enhance the capacity to utilize starch and non-cellulosic polysaccharides and promote the production of total volatile fatty acids (VFA) (43, 44). Moreover, *Prevotella* can ferment proteins and attain a N balance status in the gut (45). *Rikenellaceae_RC9_gut_group*, which is the main gene of Rikenellaceae, plays an important role in the fermentation of carbohydrates and crude protein (46, 47). Fan et al. (8) also revealed that Tibetan sheep could enhance forage degradation and fermentation by increasing the relative abundance of Bacteroidetes, *Prevotella_1*, and *Prevotellaceae_UCG-003* during the summer season compared with the winter season. Christensenellaceae are belonged to Firmicutes, which widespread in the intestines and mucous membranes of humans and animals and are important for host health due to several enzymes (48). Overall, Bacteroidetes, Firmicutes and *Prevotella* were the most dominant bacterial taxa in the rumen of Tibetan sheep, corroborating the results of previous studies on goats (49), cows (50), beef cattle (51) and yaks (52), indicating that these bacteria play a major role in immunity, health, and the ecological function of the gastrointestinal tract in ruminants. In the present study, Tibetan sheep had a strong capacity to digest protein in the feed and optimize dietary amino acid utilization with the assistance of ruminal microorganisms.

Our study showed that the ruminal metabolite composition was significantly different among the three treatment groups based on OPLS-DA analysis, suggesting that the dietary nutrient content is likely to alter the rumen metabolomic profiles of Tibetan sheep. These results were comparable with previous study of Liu et al. (53), who found that feed type could significantly change the metabolites and functional pathway of yaks. In the present study, 17 metabolic biomarkers showed significant differences between the different groups, including benzenoids, lipids and lipid-like molecules, organic acids and derivatives, organic oxygen compounds, organoheterocyclic compounds and homogeneous non-metal compounds. Notably, all 17 metabolites showed a clear increasing trend with increasing dietary protein levels. Among these metabolites, beta-Alanine and Phenylethylamine are important amino acids for animals and enriched in sheep with the higher dietary protein. Beta-Alanine is a non-essential amino acid that is synthesized in the liver tissue and is presumed to be an intermediate required for the synthesis of acrylamide and acetonitrile (54, 55). Previous studies have confirmed that beta-Alanine can improve the digestibility of soluble starch and readily fermentable carbohydrates and induce the transition from VFAs to carbohydrates (53, 56). Phenylethylamine is an essential amino acid for animals and can synthesize neurotransmitters and hormones, and participates in glucose metabolism and fat metabolism (57). In addition to these amino acids, several carbohydrates, such as D-Mannose, Allose and D-Maltose, were also enriched in the higher dietary protein groups. The main function of these carbohydrates is to provide energy for animal growth and development (58). For example, D-Mannose is an important monosaccharide for protein glycosylation in mammals and thought to promote immunity and boost energy metabolism in organisms (59, 60). KEGG analysis revealed that tryptophan metabolism was the most altered metabolic pathway among the three groups. Tryptophan metabolism is a multi-pathway and complex process that occurs in the host and its intestinal symbiotic microbiota. Several metabolites generated by the tryptophan pathway have various effects on immune function, including tryptophan metabolism in animals (61). It was found that intestinal microorganisms such as bacteria, fungi and protozoa could contribute to the formation of key tryptophan metabolites, such as anabolic D-Tryptophan in the microbial community, small peptides synthesized by fungi, indoles and their derivatives (62). The same tryptophan metabolites produced by animal intestinal bacteria are indole derivatives, such as indoleacetic acid (IAA), indole sulfuric acid (ISA), indole-3-acetaldehyde (IAAId) and tryptamine (63). In addition, tryptophan metabolites of intestinal microbes could affect host physiological health by stimulating target gene expression, modulating the mucosal immune system, and targeting specific receptors. In our study, the content of indoleacetic acid showed higher concentration in the rumen of Tibetan sheep fed the higher dietary protein level. Based on the above results, we speculate that higher dietary protein may regulate nutrient absorption and growth performance through the composition and function of ruminal metabolites.

Metabolomics is used to study the small-molecule metabolites changes in animals produced by the external disturbance and can reflect the state of physical function more accurately. Compared to microorganisms, the metabolome can reflect the most intuitive physiological state of the animal. Therefore, the rumen metabolomic profiles were more sensitive to different dietary protein levels than bacterial community compositions in the present study. Additionally, an association was found between the structure of the rumen microbiota and metabolic profiles. These results were consistent with those in donkeys (64), yaks (65), Holstein heifers (18), mice (66) and humans (67), revealing a close relationship between microbiota, metabolites and organismal health. Our study also found significant positive correlations between the dominant bacterial groups and differential metabolites using Spearman correlation analysis. Our previous study demonstrated that higher dietary protein levels could improve growth performance, carcass performance and meat quality (25). Based on these findings, it is speculated that rumen bacterial groups may promote nutrient absorption capability by positively regulating the concentration of amino acids (e.g., beta-Alanine, Phenylethylamine), carbohydrates (e.g., D-Mannose, Allose, D-Maltose, Maltulose) and organic acids (e.g., Hydroxypropanedioic acid, Indoleacetic acid, and Maleamate), and promote the nutrient absorption and growth performance of Tibetan sheep.

## Conclusions

In summary, we applied multi-omics analysis combined with microbiome and metabolomics analyses to investigate the effects of dietary protein levels on ruminal microbial communities and metabolites in Tibetan sheep. Under the same forage-to-concentrate ratio condition, the difference in dietary protein levels had no significant impact on rumen bacterial groups. Meanwhile, with increasing dietary protein levels, the concentrations of metabolites related to nutrient absorption significantly increased. In addition, the dominant microbiota was associated with different metabolites, indicating a close link between microbes and metabolites. Taking the above points into consideration, the higher protein levels (12.1 and 14.1%) were recommended as the appropriate dietary protein level in Tibetan sheep during the cold season. This study allowed us to gain a better understanding of ruminal microbial and metabolic functions and can lead to improvements in the protein level requirements within Tibetan sheep diets and nutritional regulation.

## Data Availability Statement

The datasets presented in this study can be found in online repositories. The names of the repository/repositories and accession number(s) can be found below: https://www.ncbi.nlm.nih.gov/, PRJNA801776.

## Ethics Statement

The animal study was reviewed and approved by Experimental Animal Use Ethics Committee of the Northwest Institute of Plateau Biology, Chinese Academy of Sciences.

## Author Contributions

XW, SX, and TX: conception and experiment design. XW, TX, XZ, NZ, and YG: experiment conduction. XW, HL, and XZ: statistical analysis. SK, LH, and QZ: resources. XW: writing original draft preparation. All authors have read and agreed to the published version of the manuscript.

## Funding

This work was funded by the Strategic Leading Science and Technology Program of the Chinese Academy of Sciences (XDA2005010406 and XDA23060603), Platform of Adaptive Management on Alpine Grassland-livestock System (2020-ZJ-T07), and Joint Research Project of Sanjiangyuan National Park (LHZX-2020-7).

## Conflict of Interest

The authors declare that the research was conducted in the absence of any commercial or financial relationships that could be construed as a potential conflict of interest.

## Publisher's Note

All claims expressed in this article are solely those of the authors and do not necessarily represent those of their affiliated organizations, or those of the publisher, the editors and the reviewers. Any product that may be evaluated in this article, or claim that may be made by its manufacturer, is not guaranteed or endorsed by the publisher.
