# Supplementary material for: The Response of Ruminal Microbiota and Metabolites to Different Dietary Protein Levels in Tibetan Sheep on the Qinghai-Tibetan Plateau
Source: Front Vet Sci. 2022 Jun 29;9:922817. doi: 10.3389/fvets.2022.922817 (PMC9277223; doi:10.3389/fvets.2022.922817)
Supplement: Supplementary file 1 [file Data_Sheet_1.PDF]

Supplementary Table 1. Comparisons of ruminal metabolites that significantly changed in the three treatment groups.

Groups	Metabolites	VIP	*p*-value	$\log_2$FC	Type	Metabolic classes
**HP vs LP**	beta-Alanine	1.588	0.000	-1.921	Down	Organic acids and derivatives
	Hydroxypropanedioic acid	1.553	0.001	-2.2281	Down	
	5-Hydroxyindoleacetic acid	1.522	0.001	-2.116	Down	Organoheterocyclic compounds
**HP vs MP**	D-Mannose	1.673	0.018	-0.925	Down	Organic oxygen compounds
	Allose	1.672	0.019	-0.970	Down	
	Phenylethylamine	1.550	0.007	-1.417	Down	Benzenoids
	Indan-1-ol	1.531	0.039	-1.546	Down	
	D-Maltose	1.526	0.046	-0.683	Down	Organic oxygen compounds
	Maltulose	1.508	0.039	-0.858	Down	Lipids and lipid-like molecules
**MP vs LP**	Pyrrole-2-carboxylic acid	1.938	0.000	-1.180	Down	Organoheterocyclic compounds

Indoleacetic acid	1.689	0.001	-1.604	Down	
3-Hydroxypalmitic acid	1.679	0.001	-1.075	Down	Lipids and lipid-like molecules
2,2-Dimethylsuccinic acid	1.677	0.001	-1.862	Down	
Maleamate	1.649	0.001	-1.962	Down	
3-Hydroxynorvaline	1.640	0.004	-1.230	Down	Organic acids and derivatives
Hydroxylamine	1.612	0.006	-3.163	Down	Homogeneous non-metal compounds
4-Methylcatechol	1.605	0.003	-2.454	Down	Benzenoids
